# Food allergy population thresholds: an evaluation of the dosing scheme and number of oral food challenges on the accuracy of threshold dose studies

**DOI:** 10.1186/2045-7022-5-S3-O12

**Published:** 2015-03-30

**Authors:** Rinke Klein Entink, Ben Remington, Marty Blom, Carina Rubingh, Astrid Kruizinga, Joseph Baumert, Steve Taylor, Geert Houben

**Affiliations:** 1 TNO, Zeist, The Netherlands; 2 FARRP, University of Nebraska, Lincoln, NE, USA

## 

The availability of clinically established DBPCFC minimal eliciting doses determined in food-allergic individuals is increasing and previous work has shown these data can be used to determine population based reference doses for allergen risk management. Despite the large amount of data for peanut, milk, egg and hazelnut, EU and US public health authorities have not established population thresholds for any of the allergenic foods. In the absence of regulatory guidance regarding thresholds, food producers have attempted to manage risk through the widespread application of precautionary "may contain" labelling. In turn, food-allergic consumer quality-of-life has decreased and some food-allergic individuals are ignoring these advisory statements.

This study aimed to assess the effects of the number of individual threshold doses and the dosing scheme intervals on the accuracy of estimates of population thresholds for allergenic foods. A simulation study was performed to evaluate the effects of sample size (N=20-750) and dosing scheme (derivatives of the EuroPrevall protocol) on the accuracy of the resulting threshold distribution curve. The simulations were based on clinical data on individual subject thresholds from peanut, egg and soy flour allergic individuals.

The relationships between sample size, dosing scheme and the employed statistical distribution on the one hand and the accuracy of estimation of population based eliciting doses on the other hand were obtained. The largest relative gains in accuracy were seen when sample size increases from N=20 to N=60. Additionally, proper allocation of the dosing steps is important. So, while the EuroPrevall dosing scheme is a good start, the scheme may need revision for a specific allergen as more data become available.

These results may guide risk assessors in determining minimum sample sizes for new studies and in the allocation of proper dosing schemes for allergens in provocation studies. In turn, these results will help determine dataset requirements when establishing population thresholds and reference doses leading to improved allergen risk management of food products.

